# Observation of Safety and Efficacy of Botulinum Toxin Type A in the Treatment of Tear Troughs and Mild Yelid Bags

**DOI:** 10.1111/jocd.70253

**Published:** 2025-06-04

**Authors:** Siyuan Zhou, Homgtao Liu, Zhongjie Pan, Fuqiang Pan, Qian Liang, Houhuang Qiu, Bingliang Wu, Liming Zhang, Xiang Zhou

**Affiliations:** ^1^ Department of Medical Cosmetology Second Affiliated Hospital of Guangxi Medical University Nanning City Guangxi Province China; ^2^ Guangxi Medical University Nanning City Guangxi Province China; ^3^ Guangxi Health Science College Nanning City Guangxi Province China; ^4^ The Second Affiliated Hospital of Guangxi Medical University Nanning City Guangxi Province China

**Keywords:** botulinum toxin type a, intradermal botulinum toxin injection, non‐surgical aesthetic treatment, tear trough

## Abstract

**Objective:**

To evaluate the safety and efficacy of Botulinum Toxin Type A (BTX‐A) injection for the treatment of tear troughs.

**Method:**

This study included patients with tear troughs rated as Grade 1–2 on the Barton Aesthetic Scale, who were treated between September 2023 and September 2024. These patients underwent intradermal injections of Botulinum Toxin Type A (BTX‐A). Doctors conducted pre‐ and post‐procedure assessments of the severity of tear troughs using the Barton Scale and TTRS (The Tear Trough Rating Scale). VISIA skin image analyzer (Canfield, USA) was utilized to compare the number of wrinkles, and facial scores and percentiles were calculated for objective evaluations before and after treatment. Additionally, doctors employed the Fitzpatrick Scale to assess periorbital wrinkles. This investigation was conducted as a prospective single‐center study (PSS).

**Results:**

All 42 patients completed the study. After treatment, there was a significant improvement in VISIA‐measured wrinkle count, facial scores, and percentiles. Additionally, there were significant reductions in Barton, TTRS, and Fitzpatrick scores. One case of pseudo‐eyelid bags was observed post‐treatment, while no other complications such as diplopia, lower eyelid skin laxity, or other adverse effects were reported.

**Conclusion:**

BTX‐A is effective in improving mild tear troughs while simultaneously reducing fine lines in the lower eyelid. Patients were highly satisfied with the improved appearance.

## 1 Introduction

The periorbital region is the most prone to aging signs on the face, with tear troughs being one of the most significant concerns in facial aesthetics. Tear troughs are skin grooves that initially extend from the inner canthus outwards towards the inner line of the pupil. As individuals age, these grooves gradually connect with the palpebromalar groove, distinctly separating the protruding orbital fat above from the retreating mid‐cheek area below, creating a juncture between the eyelid and cheek and imparting a baggy appearance to the lower eyelid skin [1, 2]. The formation of tear troughs is related to various factors, and there have been multiple theories about the causes of tear troughs. The current mainstream theory suggests that the occurrence of tear troughs is due to the presence of the tear trough ligament(TTL), which is a true osteocutaneous ligament located in the infraorbital region of the medial maxilla [2, 3, 4]. Based on the anatomical structure of the tear trough, surgical release of TTL used to be a commonly used method in plastic surgery for treating tear troughs. However, due to surgical trauma, complications such as hematoma and lower eyelid depression are prone to occur after surgery, leading to a long recovery period [5]. As modern life becomes increasingly fast‐paced, people's demands for beauty have also risen steadily, leading to the wider application of minimally invasive treatments in plastic surgery. The minimally invasive treatment of tear troughs, primarily involving filler injections of hyaluronic acid and autologous fat, is characterized by minimal trauma, high safety, and effectiveness. However, complications such as post‐filling deformities, the Tyndall effect, fat calcification, and embolism have not been fully resolved and require further research [6]. Other non‐surgical treatment methods also include radiofrequency, chemical peels, fractional laser, focused ultrasound, and so on [1, 7, 8]. The trend and demand for minimally invasive treatment of lower eyelid blepharoplasty is growing rapidly. BTX‐A, by blocking the transmission of nerve impulse at the neuromuscular junction, paralyzes and relaxes the muscles, and has been widely used in the field of aesthetics [9]. Its ability to adjust muscle tension and thereby achieve lifting effects in corresponding areas, such as eyebrow lifting, improvement of upper or lower facial sagging, and jawline contour enhancement, has been proven [9, 10, 11, 12]. ATWA E M and their colleagues conducted a split‐face controlled experiment using saline solution and botulinum toxin. The results showed highly significant differences between the side injected with botulinum toxin and the side injected with saline solution [13]. At present, the application of botulinum toxin in the lower eyelid is mostly combined with surgery to improve postoperative healing and scar condition, and microdroplet injection is used to reduce lower eyelid wrinkles [9, 14]. Previously, there has been no relevant research on the use of BTX‐A for tear troughs. This study aims to explore whether adjusting the orbicularis oculi muscle tension with BTX‐A and relaxing TTL at the muscular level can achieve the effects of improving tear trough deformities and reducing fine lines under the lower eyelid. The report is as follows.

## 2 Materials and Methods

### 2.1 General Information

This investigation was conducted as a PSS. A total of 42 patients with bilateral mild tear troughs treated from September 2023 to September 2024 were selected. The group consisted of male and female patients aged 18–60 years (mean age: 40.60 ± 7.20 years). The BMI of the group was 20.85 ± 1.60 kg/m^2^. Inclusion criteria for the study were as follows: (1) aged 18–60 years, regardless of gender; (2) mild tear troughs (Barton scale 1–2); (3) no prior botulinum toxin treatment within the last 6 months. Exclusion criteria included: (1) patients with a history of lower eyelid surgery; (2) allergy to BTX‐A; (3) pregnant or lactating; (4) other common contraindications to BTX‐A injections; (5) patients with known or suspected poor compliance, such as those with alcoholism, drug dependence, or mental illness.

Standardized photographic files: VISIA skin image analyzer (Canfield, USA) was used to capture images at baseline and 2 weeks, 4 weeks, and 12 weeks after treatment. All photos were taken in the original format under the same conditions and settings. Standardized views were adopted, and all photos were processed in one laboratory. Patients signed informed consent forms, and this study was approved by the Ethics Committee of the author's hospital.

### 2.2 Treatment Methods

**FIGURE 1:**
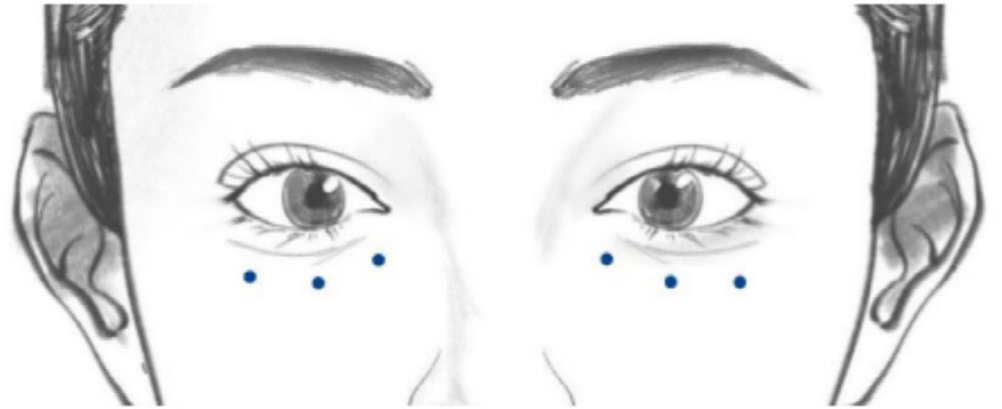
Schematic diagram of orbital injection points. Blue circles represent 0.66 units (0.01 mL) of BTX‐A.BTX‐A, Botulinum toxin type A. NS, Normal Saline.

In accordance with the experimental design, patients received injections of BTX‐A (Hengli Botulinum Toxin, Lanzhou Institute of Biological Products and Technology Co. Ltd., 100 units or 1 vial; diluted in 3 mL NS) into the lower eyelids, respectively. A 1 mL syringe with a 30G needle was used for the injections. The injection sites were located on the lower eyelids, with the injection points 1–2 mm away from the edge of the tear trough. The dose was 0.01 mL (0.66 U) per point. Depending on the length of the lower eyelid, 3 to 4 points were selected for intradermal injection, with a spacing of 7 to 8 mm between each point (Figure 1). Immediately after the injection, a skin mound could be seen, which typically disappeared within 1 to 2 h. Postoperative care included the application of an ice pack. Patients could wash their faces with water 12 h after treatment and resume usual skincare after 24 h.

### 2.3 Observation Indicators

At baseline and 2 weeks, 4 weeks, and 12 weeks after treatment, safety was assessed by recording adverse events at each stage. Efficacy was evaluated through the VISIA skin image analyzer (Canfield, USA) and assessments by two plastic surgeons. The valuation indicators included: VISIA eye wrinkle count, facial percentile, facial score [15], Fitzpatrick Classification of Perioral and Periorbital Wrinkles [16], TTRS score [17], and Barton classification [18].

#### 2.3.1 VISIA

VISIA (Canfield, USA) is a powerful skin detection instrument that can quantify and analyze the pathological features of the skin by scanning the surface and deep layers of the skin with the image of white light, ultraviolet light, and polarized light by an ultra‐high‐definition camera. The pathological features of the skin that VISIA can evaluate include skin pigmentation, pores, wrinkles, UV spots, etc. In addition to counting for each pathological feature, the VISIA skin monitor is supported by two internationally authoritative skin databases, which enable multidimensional analysis of the patient's skin, such as facial score and facial percentile. Facial scores indicate rank in the same region, age group, and race. The percentage of peers whose current facial condition is surpassed by the individual's facial state is indicated by the facial score percentile [15].

#### 2.3.2 Fitzpatrick Classification of Perioral and Periorbital Wrinkles

FITZPATRICK designed a clinical scoring system to evaluate the degree of wrinkles and photodamage during the study on the clinical improvement of pulsed CO_2_ laser treatment for perioral and periorbital wrinkles. The scoring system divided facial wrinkles and photodamage into three levels, with a score range of 0–9 points. This grading standard takes into account the depth of wrinkles and the degree of degeneration of elastic tissue, which is of great significance in the evaluation of wrinkles around the orbit and mouth, and has been widely used [16].

#### 2.3.3 TTRS Score

TTRS is one of the most commonly used scales designed and developed by Sadick to assess the shape of the lower eyelid. TTRS is a scoring system that measures the distance from the anterior lacrimal crest to the depth of the trough, with 1 point per millimeter of depth. In addition, hyperpigmentation is also included in the scoring criteria, with 1 point for no hyperpigmentation, 2 points for mild hyperpigmentation, 3 points for moderate hyperpigmentation, and 4 points for strong or deep hyperpigmentation. Lower eyelid skin rhytidosis is also graded from 1 to 4, representing mild, moderate, advanced, and severe. Prolapse of nasal fat pockets accentuates the depth of the trough and is rated as mild (1 point), moderate (2 points), or severe (3 points). The scale helps doctors and patients objectively assess the severity of lacrimal malformations and select appropriate treatment options [17].

#### 2.3.4 Barton Classification

Barton grading, first proposed in 2004, is a classification system designed by Barton et al. based on anatomical features, which is divided into 0–4 grades according to the degree of the apparent extent of the tear trough, length, and depth of depressions. It has been widely used in the evaluation of tear troughs [18]. Grade 0: Absence of medial or lateral demarcation lines at the arcus marginalis or orbital rim, with preservation of a smooth youthful contour and no visible transition zone at the orbit‐cheek junction. Grade 1: Mild presence of a subtle medial demarcation line or shadow, while maintaining a smooth lateral transition at the lid‐cheek junction. Grade 2: Moderately prominent visible demarcation of the lid‐cheek junction extending continuously from medial to lateral aspects. Grade 3: Severe demarcation at the orbit‐cheek junction characterized by a distinct step deformity between the orbital and cheek compartments.

### 2.4 Statistical Analysis

The data obtained from this study were statistically analyzed using SPSS 24.0. Count data were expressed as cases, and comparisons were made using the *χ* ^2^ test. Measurement data were expressed as mean ± standard deviation ($\bar{x}$ ± s), and comparisons before and after treatment within the group were made using the *t*‐test. A *p*‐value of < 0.05 was considered statistically significant.

## 3 Results

All 42 patients in this study completed the 3‐month follow‐up. All patients had no underlying diseases.

### 3.1 VISIA

VISIA assesses the severity of wrinkles by measuring the average roughness (Ra) and quantifies the number of wrinkles, calculating facial scores and percentiles [15]. Prior to treatment, the number of periorbital wrinkles, facial scores, and percentiles were measured for all patients. The wrinkle count around the eyes of all patients before treatment was (left: 160.75 ± 29.97, right: 161.71 ± 25.65). The wrinkle count around the eyes of the patients at the second, fourth, and twelfth weeks after treatment was (left: 93.71 ± 32.48, right: 96.95 ± 25.70) (left: 87.33 ± 32.06, right: 99.67 ± 23.41) and (left: 85.67 ± 18.41, right: 97.36 ± 24.04). The number of periorbital frontal wrinkles was significantly reduced from the second week after treatment, and the difference was statistically significant compared with the baseline, and this trend was maintained until the 12th week after treatment (Tables 1, 2 and Figure 5).

**TABLE 1:** Wrinkle count (left).

Follow‐up time	Wrinkle count	*p*
0 w	160.57 ± 29.97	
2 w	93.71 ± 32.48	< 0.0001
4 w	87.33 ± 32.06	< 0.0001
12 w	85.67 ± 18.41	< 0.0001

**TABLE 2:** Wrinkle count (right).

Follow‐up time	Wrinkle count	*p*
0 w	161.71 ± 25.65	
2 w	96.95 ± 25.70	< 0.0001
4 w	99.67 ± 23.41	< 0.0001
12 w	97.36 ± 24.04	< 0.0001

Before treatment, the patients' VISIA facial scores and facial percentiles were (left: 48.99 ± 14.72, right: 46.14 ± 10.49) and (left: 22.64 ± 11.93, right: 24.81 ± 8.34). Two weeks after treatment, the VISIA facial scores and facial percentiles were (left: 24.55 ± 14.72, right: 31.93 ± 10.51) and (left: 62.33 ± 15.48, right: 46.71 ± 8.86). Compared with baseline 2 weeks ago, the patient's facial scores and facial percentiles were significantly improved, and the difference was statistically significant (*p* < 0.05). The VISIA facial score (left: 24.24 ± 11.29, right: 31.64 ± 8.64) and facial percentile (left: 62.76 ± 15.16, right: 50.76 ± 14.06) at week 4 were not significantly different from the VISIA facial score (left: 24.53 ± 11.47, right: 30.03 ± 8.44) and facial percentile (left: 62.64 ± 15.48, right: 48.38 ± 14.38) at week 12 (Tables 3, 4, 5, 6).

**TABLE 3:** VISIA facial scores (left).

Follow‐up time	VISIA facial scores	*p*
0 w	48.99 ± 14.72	
2 w	24.55 ± 11.47	< 0.0001
4 w	24.24 ± 11.29	< 0.0001
12 w	24.53 ± 11.47	< 0.0001

**TABLE 4:** VISIA facial scores (right).

Follow‐up time	VISIA facial scores	*p*
0 w	46.14 ± 10.49	
2 w	31.93 ± 10.51	0.0001
4 w	31.67 ± 8.64	< 0.0001
12 w	30.03 ± 8.44	< 0.0001

**TABLE 5:** VISIA facial percentiles (left).

Follow‐up time	VISIA facial percentiles	*p*
0 w	22.64 ± 11.93	
2 w	62.33 ± 15.48	< 0.0001
4 w	62.76 ± 15.56	< 0.0001
12 w	62.64 ± 15.16	< 0.0001

**TABLE 6:** VISIA facial percentiles (right).

Follow‐up time	VISIA facial percentiles	*p*
0 w	24.81 ± 8.34	
2 w	46.71 ± 8.86	< 0.0001
4 w	50.76 ± 14.06	< 0.0001
12 w	48.83 ± 14.38	< 0.0001

**TABLE 7:** Fitzpatrick classification.

Follow‐up time	Fitzpatrick classification			*p*
	Grade			
	1	2	3	
0 w	11	21	10	
2 w	33	9	0	< 0.001
4 w	35	7	0	< 0.001
12 w	30	12	0	< 0.001

**TABLE 8:** TTRS score.

Follow‐up time	TTRS score	*p*
0 w	7.21 ± 1.11	
2 w	3.26 ± 0.91	< 0.0001
4 w	3.45 ± 1.04	< 0.0001
12 w	3.76 ± 0.73	< 0.0001

**TABLE 9:** Barton classification.

Follow‐up time	Barton classification				*p*
	Grade				
	0	1	2	3	
0 w	0	2	40	0	
2 w	4	28	9	1	< 0.001
4 w	2	34	5	1	< 0.001
12 w	2	32	7	1	< 0.001

**FIGURE 2:**
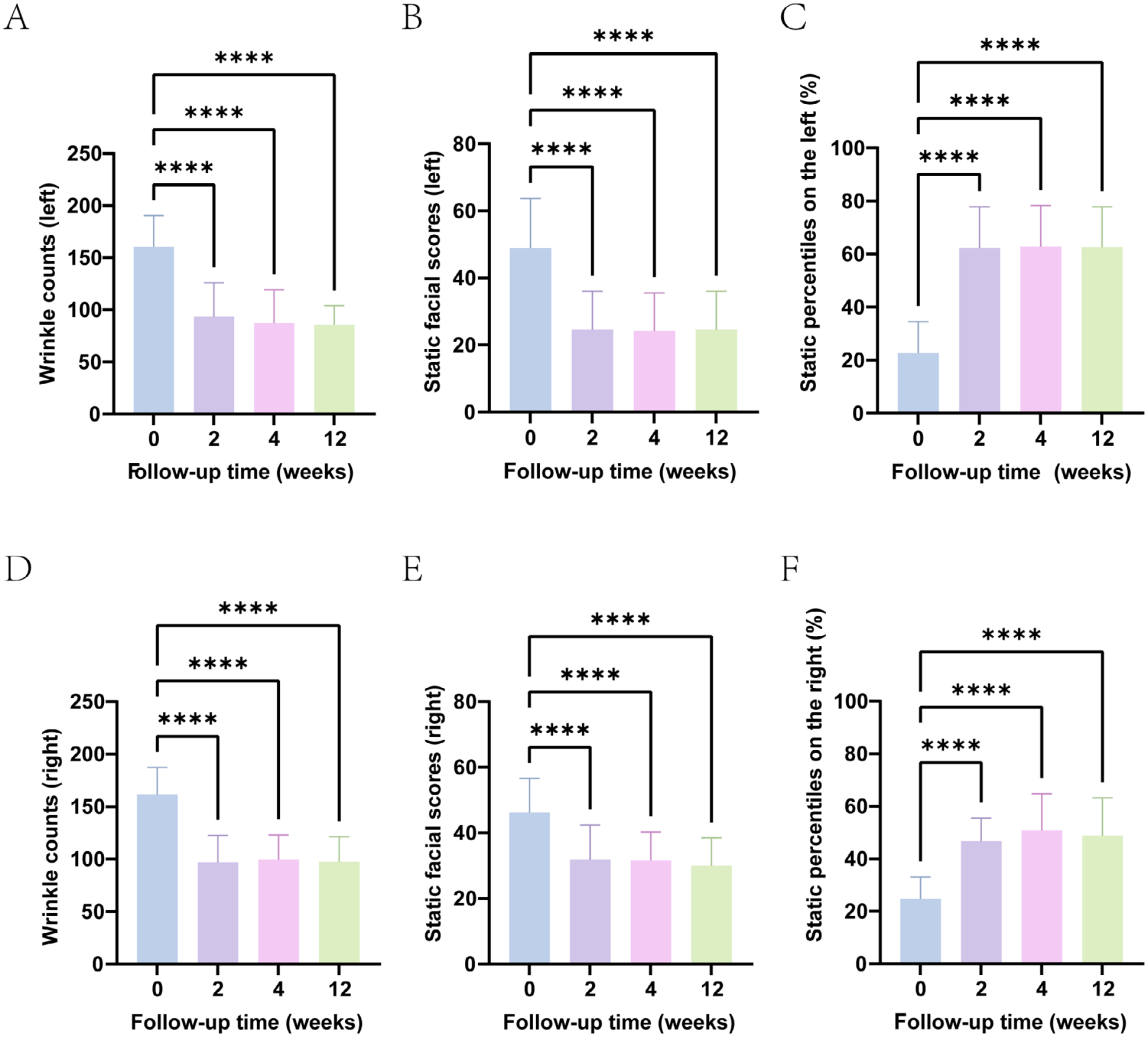
Evaluation of the degree of improvement was conducted using VISIA wrinkle count, percentiles, and facial scores before treatment and at 2 weeks, 4 weeks, and 12 weeks after treatment. (A) Count of wrinkles around the left eye; (B) VISIA Facial score on the left side; (C) VISIA Percentile of the left; (D) Count of wrinkles around the right eye; (E) VISIA Facial score on the right side; (F) VISIA Percentile of the right facial. *p < 0.05, **p < 0.01, ***p < 0.001, ****p < 0.0001.

The results showed that patients treated with botulinum toxin could reduce the fine lines of the lower eyelid as early as 2 weeks after treatment. At the follow‐up of the fourth and twelfth weeks, although the statistical results of VISIA were different, there was no significant difference (*p* > 0.05), indicating that the improvement effect of botulinum toxin on the lower eyelid could continue to 12 weeks after treatment (Figure 2).

### 3.2 Fitzpatrick Classification of Perioral and Periorbital Wrinkles

The Fitzpatrick Wrinkle Score system was used to assess the severity of perioral and periorbital wrinkles [16]. All patients were evaluated by 2 plastic surgeons before treatment. The number of patients with Fitzpatrick grade 1 and 2 increased significantly at 2, 4, and 12 weeks after treatment. The results showed that this method could improve the fine lines of the lower eyelid, and the effect lasted for 12 weeks after treatment (Table 7).

### 3.3 TTRS **Score**

**FIGURE 3:**
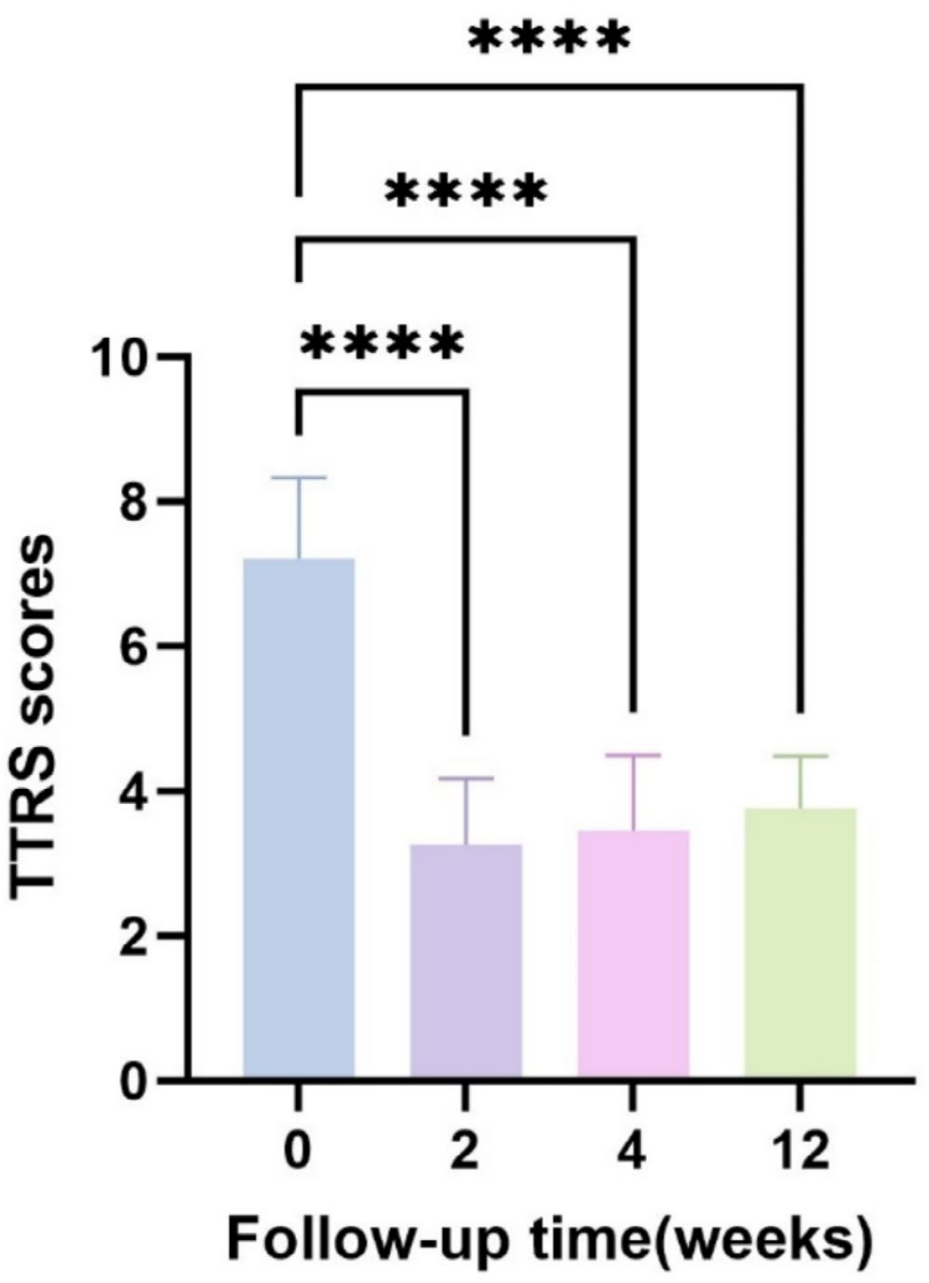
TTRS score was used to evaluate the improvement of tear trough at 0 week and at 2, 4, and 12 weeks after treatment. *p < 0.05, **p < 0.01, ***p < 0.001, ****p < 0.0001.

TTRS is an effective and reproducible method for assessing tear troughs, with minimal inter‐observer variability [17]. The mean baseline TTRS score was 7.21 ± 1.11. After 2 weeks of treatment, the TTRS score decreased to 3.26 ± 0.91. The score was 3.45 ± 1.04 after 4 weeks of treatment, and 3.76 ± 0.73 after 12 weeks of treatment. There was no significant difference between the second, fourth, and twelfth weeks of treatment (*p* > 0.05). In other words, this method can improve the tear trough, and the effect can last for 12 weeks. (Table 8, Figure 3).

### 3.4 Barton Classification

**FIGURE 4:**
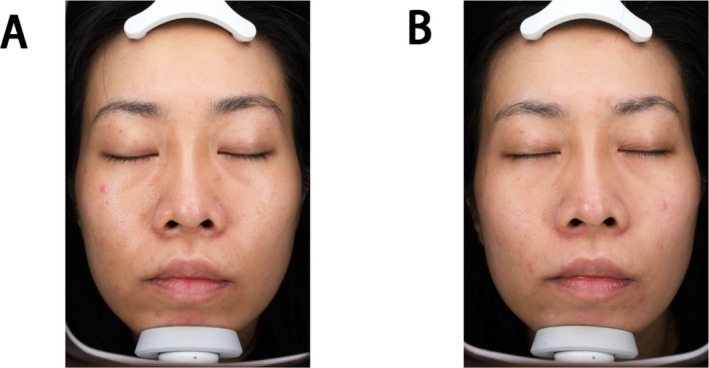
A female, 38 years old, before treatment (A), TTRS score: 2‐mm depth, no hyperpigmentation, moderate fat prolapse, and moderate lower eyelid skin rhytidosis = 7; Barton classification: Grade II; Fitzpatrick classification: Grade II；(VISIA, right) facial scores: 26.186, facial percentiles: 35%, (VISIA, left) facial scores: 23.309, facial percentiles: 48%. 12 weeks after treatment (B), TTRS score: 1‐mm depth, no hyperpigmentation, mild fat prolapse, and mild lower eyelid skin rhytidosis = 4; Barton classification: Grade I; Fitzpatrick Classification: Grade I, (right) VISIA facial scores: 23.209, facial percentiles: 40%, (left) VISIA facial scores: 18.623, facial percentiles: 70%.

Barton classification is a widely used aesthetic scale to evaluate the lower eyelid from the perspective of anatomy, which classifies and grades the aging of the tear trough [18]. Similarly, the number of patients in the experimental group was rated as Barton's grade by two plastic surgeons before treatment and after 2, 4, and 12 weeks of treatment. There was a significant increase in the number of patients who were evaluated as grade 0 and grade 1 in the second week after treatment. The difference was statistically significant compared with baseline (*p* < 0.05) (Table 9, Figure 4).

**FIGURE 5:**
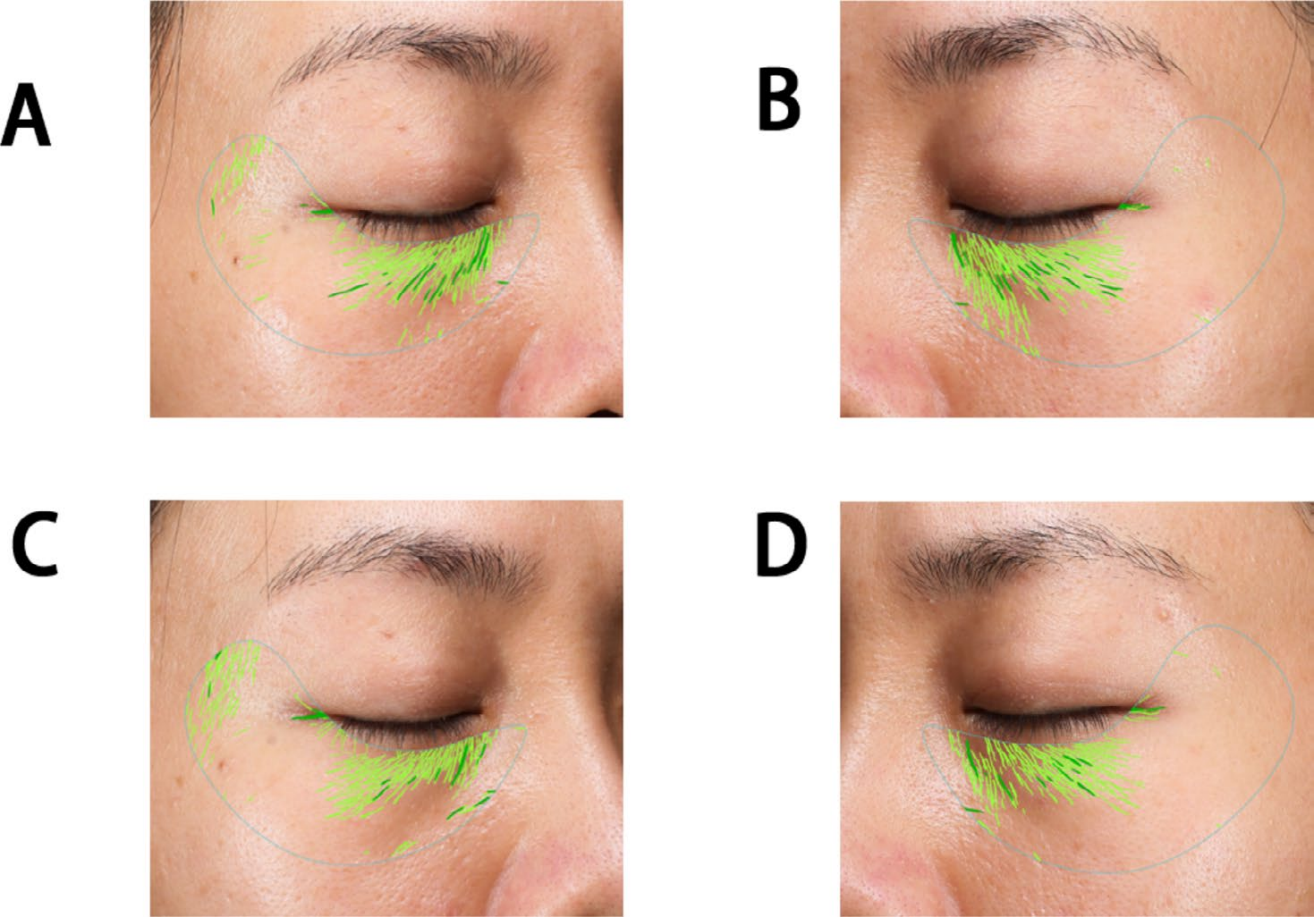
Improvement of fine lines in the lower eyelid: (A) Right eye before treatment, wrinkle count: 116; (B) Left eye before treatment, wrinkle count: 78; (C) Right eye 12 weeks after treatment, wrinkle count: 114; (D) Left eye 12 weeks after treatment, wrinkle count: 69.

### 3.5 Adverse Events

**FIGURE 6:**
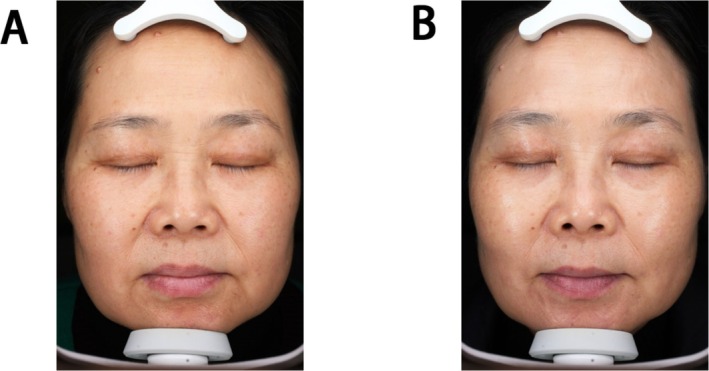
A female, 52 years old, before treatment (A), TTRS score: 1‐mm depth, no hyperpigmentation, mild fat prolapse, and mild lower eyelid skin rhytidosis = 4; Barton classification: Grade I; Fitzpatrick classification: Grade I; (VISIA, right) wrinkle count: 56, facial scores: 19.133, facial percentiles: 82%, (VISIA, left) wrinkle count: 84, facial scores: 23.309, facial percentiles: 48%. 2 weeks after treatment (B), the patient developed false bags under the lower eyelid. TTRS score: 1‐mm depth, mild hyperpigmentation, moderate fat prolapse, and moderate lower eyelid skin rhytidosis = 7; Barton classification: Grade II; Fitzpatrick classification: Grade II; (VISIA, right)wrinkle count: 92, facial scores: 22.692, facial percentiles: 73%, (VISIA, left) wrinkle count: 79, facial scores: 23.469, facial percentiles: 45%.

Immediately after injection, all patients exhibited visible skin mounds, which spontaneously resolved within 1 to 2 h post‐treatment. One patient developed a pseudo lower eyelid bags 2 weeks after injection (Figure 5). The increase in wrinkles on the lower eyelid was considered to be caused by pseudo lower eyelid bags. There were no reports of bruising, lower eyelid margin relaxation, double vision, or dry eye symptoms (Figure 6).

## 4 Discussion

The skin of the eyelids is one of the thinnest areas of facial skin [14, 19], and the anatomy around the orbit is complex, making it more prone to aging and deformation. The appearance of tear troughs and eyelid bags can give the face a fatigued look. Plastic surgeons have proposed several explanations and hypotheses regarding the formation of tear troughs: 1. Caused by the descent and traction of the malar fat pad; 2. “Indentations” created by the attachment of the orbital septum to the orbital rim; 3. Fat loss in the tear trough area or protrusion of orbital fat above the tear trough; 4. It is the convergence point of the orbicularis oculi muscle, levator labii superioris alaeque nasi muscle, and levator labii superioris muscle; 5. “Cracks” between the palpebral and orbital portions of the orbicularis oculi muscle, etc. [4]. The importance of the anatomical structure of TTL in the formation of tear troughs has now been clearly established [2].

Releasing TTL and related surgical procedures can significantly improve the shape of the lower eyelids and are commonly used for moderate to severe tear troughs and eyelid bag changes. However, surgery is invasive and requires a long recovery period. Non‐surgical treatments include hyaluronic acid fillers, autologous fat transplantation, as well as emerging new options such as radiofrequency, laser, and focused ultrasound therapy [1, 7, 8]. Each method has its own advantages and specific indications, but fillers are the mainstream method for patients with mild tear troughs. However, their disadvantages include adverse reactions such as displacement and malformation after filling, Tyndall effect, vascular embolism, tissue necrosis, pain, scarring, and pigmentation changes [6].

According to the American Society of Plastic Surgeons, since the Food and Drug Administration (FDA) approved the use of botulinum toxin for cosmetic purposes, it has become the most commonly used minimally invasive cosmetic procedure [20]. While treating blepharospasm with Botox Type A, Jean Carruthers accidentally discovered that the toxin could eliminate wrinkles around the patient's eyes. Later, together with Alastair Carruthers, they introduced this finding into the field of aesthetics and achieved satisfactory results, leading to its approval for treating moderate to severe glabellar frown lines [21, 22, 23, 24]. Since then, the application of Botox Type A has continued to expand, and BTX‐A is now widely used for full‐face wrinkle reduction. Numerous articles have reported the use of BTX‐A to adjust muscle tension, achieving effects such as eyebrow lifting, jawline enhancement, eye opening enlargement, and neck wrinkle reduction [9, 10, 11, 12, 25]. However, due to the superficial nature of the treatment in the lower eyelid skin and the orbicularis oculi muscle, injecting BTX‐A can easily lead to related complications such as lower eyelid skin laxity, eye bag protrusion, orbicularis oculi muscle dysfunction, and even diplopia [25, 26]. Treatment of the lower eyelid is considered an advanced technique in botulinum toxin therapy, and currently, the application of BTX‐A in the lower eyelid is relatively limited [9, 14].

Injection therapy has become very common in facial rejuvenation treatments. All patients in this study developed skin mounds post‐injection that spontaneously resolved within 1–2 h, with no documented cases of localized edema throughout the observation period. Although BTX‐A is considered relatively safe, it remains associated with potential adverse events. Commonly encountered short‐term complications of BTX‐A injections include procedure‐related pain, localized edema, ecchymosis, and transient hypoesthesia, with some individuals also reporting immediate post‐procedural cephalalgia of limited duration [27]. To minimize the occurrence of short‐term complications such as ecchymosis and edema, preventive measures can be implemented including pre‐procedural local anesthesia, adherence to precision injection techniques utilizing anatomical landmarks, and peri‐injection cryotherapy. To minimize the occurrence of short‐term complications such as ecchymosis and edema, preventive measures can be implemented including pre‐procedural local anesthesia, adherence to precision injection techniques utilizing anatomical landmarks, and peri‐injection cryotherapy. Long‐term complications may encompass diplopia, unilateral oral commissure ptosis, paralytic ectropion, pseudo lower eyelid bags, and dry eye syndrome. Diplopia and oral commissure ptosis specifically result from BTX‐A diffusion into deeper muscular layers beyond the targeted zones, typically involving extraocular muscle involvement or unintended neuromuscular blockade of facial expression muscles [25, 28]. BTX‐A administration in the lower eyelid may induce excessive relaxation of the orbicularis oculi muscle, resulting in pseudo lower eyelid bags, lower lid laxity, or ectropion formation. These biomechanical alterations thereby elevate the risk of exposure keratitis due to compromised ocular surface protection. Furthermore, neurotoxin spread may also precipitate dry eye syndrome through diminished blink reflex efficiency and reduced lacrimal pump function mediated by presynaptic acetylcholine release inhibition at the neuromuscular junctions [25]. Therefore, the injection depth, dosage, and unit precision of BTX‐A in the periocular region are of critical importance.

BTX‐A is commonly used in the lower eyelid to ameliorate wrinkles; however, owing to the intricate anatomical complexity of the periorbital region, one of the most frequent adverse events encountered during its application is the formation of pseudo lower eyelid bags. The occurrence of adverse events was closely related to the injection site and dose. The traditional injection point of the lower eyelid is located at the intersection of the midline of the pupil and 3 mm below the ciliary margin, and the dose is mostly 1–2 U, but it still easily leads to the appearance of pseudo lower eyelid bags [20]. This phenomenon arises from BTX‐A‐induced relaxation of the orbicularis oculi muscle and subsequent reduction in muscular tone, leading to progressive impairment of its restrictive effect on the orbital septum fat, ultimately resulting in the appearance or exacerbation of lower eyelid bags [3]. Consequently, this methodology is recognized as a high‐risk procedure in BTX‐A therapeutics. Robert E. Clark conducted a study injecting varying doses of BTX‐A 3 mm below the ciliary margin to validate its efficacy in improving lower eyelid rhytides and achieving orbital fissure enlargement. The investigation revealed that escalating doses were significantly correlated with increased incidence of adverse events, including dry eye syndrome and pseudo lower eyelid bags [25]. Xing Fan et al. employed a microdroplet technique for precision injections in the lower eyelid, demonstrating effective improvement of infraorbital rhytides while achieving complete absence of pseudo lower eyelid bags adverse events [14]. Therefore, we chose the microdrop injection method and injected only at the edge of the lacrimal groove, which effectively limited BTX‐A's therapeutic spread while maintaining efficacy. Among all 42 treated cases, only one patient (2.3%) developed pseudo lower eyelid bags.

Wong et al. clearly demonstrated through dissection of 48 cadavers that the prominence of the tear trough anatomically originates from TTL [2]. This ligament extends from the insertion point of the medial canthal tendon to approximately the medial pupillary line, then continues laterally as the orbicularis oculi retaining ligament. TTL originates from the maxilla and densely inserts into the skin at the precise location of the tear trough, making it an osteocutaneous ligament. Wrinkles in the lower eyelid skin indicate that the palpebral and orbital portions of the orbicularis oculi muscle move upward during squinting [2]. Therefore, in this study, we attempted to release TTL at the muscular level and improve the tear trough by altering the tension of the orbicularis oculi muscle.

Intradermal, subcutaneous, and intramuscular injection are three commonly used injection techniques. The dermis is a dense fibrous connective tissue, and theoretically, the injection and dissemination of BTX‐A into the skin are more resistant than those of subcutaneous injection [29]. The skin of the eyelid is almost the thinnest part of the whole body [19]. In order to avoid affecting deeper muscles and leading to adverse events such as diplopia, we chose intradermal injection rather than orbicularis oculi or subcutaneous injection. At present, studies have shown that the effective area of intradermal injection is significantly smaller than that of subcutaneous injection [29].

The injection dose of botulinum toxin type A (BTX‐A) constitutes a critical determinant for both therapeutic efficacy and duration. While the longevity of effect demonstrates dose‐dependency, excessive dosing may induce prolonged stiffness, discomfort, and adverse events including blepharoptosis. Microdrop injection refers to the BTX‐A multi‐point, small dose injection into the dermis, microdrop injection with relatively low concentration, more shallow injection method to get a good cosmetic effect, while to a certain extent to make up for the defects of traditional injection technology, that is, after the injection of facial muscle stiffness, stiff expression not natural so‐called “poker face”. While improving the face, microdrop injection avoids the influence on the expression as much as possible, and the injection effect is more natural. Robert E Clark's research results showed that single point injection of BTX at the lower eyelid could improve the lower eyelid fold and increase the eyelid aperture. On the basis of this research, they evaluated the application measurement of BTX‐A at the lower eyelid. They injected a total of 2 U, 4 U, and 8 U of BTX‐A subcutaneously 3 mm below the ciliary margin of the middle and lateral pupillary line, and observed dose‐dependent effects. They found that increasing doses were associated with side effects, including scleral exposure, photophobia, and lower eyelid edema [25]. Steinsapir et al. injected BTX‐A into the bilateral eyebrow and glabellar region of 227 patients by micro‐drop treatment, with 0.33–0.66 U per point. The injection point was not lower than the lower edge of the eyebrow, and the depth was the dermis. Image analysis software was used to quantitatively analyze the curative effect of patients before and after injection, which proved that this method could effectively reduce the glabellar wrinkles and forehead wrinkles and better preserve the expression ability of the eyebrow and forehead compared with the traditional injection method [30]. In order to ensure the width of the effect of botulinum toxin on the lower eyelid and avoid affecting deeper muscles, this study chose to control the total amount of botulinum toxin at 2–3 U, split‐point injection, and 0.66 U per point. In order to ensure the width of the action of botulinum toxin in the lower eyelid and avoid affecting the deeper muscles, we choose to shorten the interval distance of 0.7–0.8 cm and use small doses of intradermal multi‐point injection. This method effectively improved mild tear troughs, enhanced the grading composition of lower eyelid scores, reduced the incidence of complications associated with lower eyelid fillers, and minimized fine lines in the lower eyelid. Among the 42 patients, only one case of pseudo‐eye bag was observed, with no other complications such as pseudo‐eye bags, diplopia, or lower eyelid laxity occurring in the remaining patients.

The evaluation of therapeutic effect in aesthetic medicine is often subjective and requires a variety of indicators to evaluate the outcome of treatment. In this paper, VISIA was used to quantitatively and objectively evaluate the effectiveness of this method in the treatment of tear trough, and at the same time, it was clear that this method was effective in improving the fine lines of the lower eyelid. In addition, we combined the evaluation of non‐treating doctors to conduct quantitative analysis to determine the effectiveness of the method and ensure the accuracy of the results.

The sample size of this study is small (42 cases), and the sample size should be further expanded, and the split‐plane experiment can be conducted later. Generally, the action time of BTX‐A is between 3 and 6 months, and the experimental follow‐up time of 3 months is mainly due to the consideration of a small injection dose and superficial level, but the experimental results show that this method continues to be effective at 3 months, and our next experiment can appropriately extend the follow‐up time. This is a single‐center trial, only for Asian races, and it should be expanded to see if there are differences in efficacy between different regions and races. Further study of this experiment is necessary; we need to find the best dose for different degrees of tear trough, as well as the time to reach the peak effect and the best dose for lasting effect. These efforts will provide clinical support for the safety and standardization of this treatment.

## 5 Conclusions

For patients with mild tear trough deformity who are not candidates for or decline surgical/dermal filler interventions, BTX‐A therapy demonstrates measurable improvement in lower eyelid aesthetic contour and reduction of infraorbital rhytides. This study provides a beneficial exploration and research for the non‐surgical treatment of tear groove and pouch, which has clinical guiding significance and is worthy of clinical promotion. This exploratory study introduces a novel therapeutic option for patients with mild tear trough deformity. While BTX‐A demonstrates periocular rejuvenation effects, its therapeutic limitations persist due to incomplete release of TTL dermal anchoring mechanism. The clinical necessity for repeated injections further underscores the modality's temporal constraints. Different treatment methods have their own advantages and disadvantages. The combination of minimally invasive methods may be the direction of future development, and further research is needed to provide patients with reliable efficacy, safety, minimally invasive, and less painful treatment options.

## Author Contributions

Siyuan Zhou participated in the writing and revision of the paper. Homgtao LIU and Zhongjie PAN participated in the analysis, interpretation and data collection. Houhuang QIU and Fuqiang Pan, and Qian Liang assisted with operation, data collection, analysis, and interpretation; Liming Zhang and Xiang Zhou contributed to the drafting, revision, and publication approval of the manuscript.

## Ethics Statement

This project fully considered and protected the rights and interests of the study objects. It meets the criteria of the Ethical Review Committee. This study has passed ethical review [No. 2023‐KY(0772)].

## Consent

Consent for publication: All authors approved the final manuscript and the submission to this journal. Written informed consent for publication was obtained from all participants.

## Conflicts of Interest

The authors declare no conflicts of interest.

## Supporting information


Data S1.

## Data Availability

The data that support the findings of this study are available on request from the corresponding author. The data are not publicly available due to privacy or ethical restrictions.
